# Emergence of high colistin resistance in carbapenem resistant *Acinetobacter baumannii* in Pakistan and its potential management through immunomodulatory effect of an extract from *Saussurea lappa*

**DOI:** 10.3389/fphar.2022.986802

**Published:** 2022-09-16

**Authors:** Umaira Ahsan, Fizza Mushtaq, Sidrah Saleem, Abdul Malik, Hira Sarfaraz, Muhammad Shahzad, Bernt Eric Uhlin, Irfan Ahmad

**Affiliations:** ^1^ Institute of Biomedical and Allied Health Sciences, University of Health Sciences, Lahore, Pakistan; ^2^ Department of Microbiology, University of Health Sciences, Lahore, Pakistan; ^3^ Department of Pharmacology, University of Health Sciences, Lahore, Pakistan; ^4^ Department of Molecular Biology and Umeå Centre for Microbial Research (UCMR), Umeå University, Umeå, Sweden

**Keywords:** colistin, multiple drug resistence, *Acinetobacter baumannii*, doxycycline, *saussurea lappa*

## Abstract

Carbapenem resistant *Acinetobacter baumannii* has emerged as one of the most difficult to treat nosocomial bacterial infections in recent years. It was one of the major causes of secondary infections in Covid-19 patients in developing countries. The polycationic polypeptide antibiotic colistin is used as a last resort drug to treat carbapenem resistant *A. baumannii* infections. Therefore, resistance to colistin is considered as a serious medical threat. The purpose of this study was to assess the current status of colistin resistance in Pakistan, a country where carbapenem resistant *A. bumannii* infections are endemic, to understand the impact of colistin resistance on virulence in mice and to assess alternative strategies to treat such infections. Out of 150 isolates collected from five hospitals in Pakistan during 2019–20, 84% were carbapenem resistant and 7.3% were additionally resistant to colistin. There were two isolates resistant to all tested antibiotics and 83% of colistin resistant isolates were susceptible to only tetracycline family drugs doxycycline and minocycline. Doxycycline exhibited a synergetic bactericidal effect with colistin even in colistin resistant isolates. Exposure of *A. baumannii* 17978 to sub inhibitory concentrations of colistin identified novel point mutations associated with colistin resistance. Colistin tolerance acquired independent of mutations in *lpxA, lpxB, lpxC*, *lpxD*, and *pmrAB* supressed the proinflammatory immune response in epithelial cells and the virulence in a mouse infection model. Moreover, the oral administration of water extract of *Saussuria lappa,* although not showing antimicrobial activity against *A. baumannii in vitro*, lowered the number of colonizing bacteria in liver, spleen and lung of the mouse model and also lowered the levels of neutrophils and interleukin 8 in mice. Our findings suggest that the *S. lappa* extract exhibits an immunomodulatory effect with potential to reduce and cure systemic infections by both opaque and translucent colony variants of *A. baumannii.*

## Introduction

*Acinetobacter baumannii* is an emerging nosocomial pathogen responsible for a diverse range of infections, including blood stream infections, meningitis, urinary tract infections and ventilator-associated pneumonia (Rice, 2008). Bacteremia and acute respiratory distress syndrome associated with *A. baumannii* infections are often fatal (Mcconnell et al., 2013). Over the last 2 decades, a tremendous rise in antimicrobial resistance of *A. baumannii* has been reported throughout the world (Antunes et al., 2014; Ayoub Moubareck and Hammoudi Halat, 2020; Karah et al., 2020; Khalid et al., 2020; Ibrahim et al., 2021). In contrast, new additions to the arsenal of useful antimicrobial drugs are remarkably low. Consequently, the spectrum of available drugs for treatment is being narrowed down (Kempf and Rolain, 2012). The carbapenems have been in frequent use to treat infections caused by Gram-negative rods, but resistance to this class of drugs has been rapidly observed in *Acinetobacter* species (Paterson, 2006).

Pakistan stands among countries where carbapenem resistant *A. baumannii* are reported at very high frequency (Begum et al., 2013; Evans et al., 2011; Hasan et al., 2014; Hsu et al., 2017; Karah et al., 2020; Khalid et al., 2020). Carbapenem resistant *A. baumannii* infections are challenging to treat due to limited available options. Colistin is considered as an antibiotic of last option for the treatment of carbapenem resistant *A. baumannii* (Isler et al., 2019).

Colistin is a cationic polypeptide antibiotic that consists of a cyclic decapeptide joined by an α-amide linkage to a fatty acyl chain. With a single amino acid difference to polymyxins B, both antibiotics exhibit similar anti-microbial activities against Gram-negative bacteria (Gallardo-Godoy et al., 2016; Poirel et al., 2017). Polymyxins directly target bacterial outer membranes and exert the antibacterial effect by a two-step mechanism. In the first step, the positively charged antibiotic binds to negatively charged lipopolysaccharide. The LPS-polymyxin interactions cause an electrostatic effect leading to the permeabilization of the outer membrane followed by its destabilization (Poirel et al., 2017). Any alteration in lipid A bilayer that nullifies its charge can prevent its binding to colistin. Thus, the distinct mechanism of resistance usually involves alterations to lipid A that reduces or nullifies the charge-based interaction with polymyxins. In a number of Gram-negative bacteria, the modification of lipid A by addition of 4-amino-4-deoxy-l-arabinose (l-Ara4N) and/or phospho ethanolamine (PEtn) reduces the net LPS negative charge to decrease the LPS-polymyxin interactions. In *A. baumannii*, modifications of LPS, in order to achieve colistin resistance is mainly achieved by point mutations in two component system *pmrAB* that cause an addition of phospho ethanolamine to LPS (Moffatt et al., 2010a). Alternatively, loss of LPS due to mutations in LPS biogenesis machinery involving loci *lpxA, lpxC,* and *lpxD* also lead to colistin resistance (Moffatt et al., 2010a).

Here, we focus on the status of colistin usage in Pakistan, a country where carbapenem resistant *A. baumannii* infections are endemic, by analysing the impact of colistin resistance on virulence and inflammation in a mouse model. We evaluate the potency of second line drug doxycycline against colistin resistance isolates and assess the efficacy of *Saussurea lappa* extracts against inflammation caused by *A. baumannii*.

## Results

### High frequency of colistin resistance in extreme drug resistant isolates of *Acinetobacter baumannii* in Pakistan

Previously, we reported on the molecular epidemiology and drug resistant features of *A. baumannii* isolates collected from hospitals in Pakistan during 2013–2015. No colistin resistance was found among those isolates (Karah et al., 2020). In the follow up, here we report the current status of colistin resistance in isolates collected from five hospitals in Pakistan during 2019–2020. For this study, a collection of 150 clinical isolates was obtained. Out of the 150 clinical isolates, 28 (19.4%) were from the respiratory tract infections, 56 (38.8%) from the blood or central venous catheter, 23 (16.3%) from the wound infections and 36 (25%) from the urinary tract infections. The isolates showed resistance rates of 100% (150/150) to ampicillin/sulbactam, piperacillin/tazobactam, cefepime, cefotaxime, ceftazidime, ceftriaxone, gentamicin, tobramycin, ciprofloxacin, levofloxacin, and trimethoprim/sulfamethoxazole. In addition, 84% (126/150) of the isolates were resistant to imipenem, 9,3% (14/150) to minocycline and doxycycline, and 7,3% (11/150) to colistin and polymyxin B (Table 1). Except for the findings on colistin resistance, data were in line with previous studies describing extensive occurrence of multidrug-resistant strains of *A. baumannii* in Pakistan (Begum et al., 2013; Khalid et al., 2020). More alarmingly, there were two isolates resistant to all tested 2^nd^ line and 3^rd^ line antibiotics including colistin, moxifloxacin and doxycycline. Both of these isolates (2204 and 2208) were obtained from tracheal secretion of persons admitted to two different hospitals in 2020 and belong to OXA-66 type of strains with OXA-51 beta lactamase (Table 2). The MIC of colistin varied from strain to strain in colistin resistant isolate with a range between 8 ug/ml to 32 ug/ml. Nine out of eleven colistin resistant isolates were hetero resistant to all tested antibiotics except tetracycline drugs doxycycline and minocycline whereas the remaining two isolates were resistant to all tested antibiotics.

**TABLE 1:** Frequency of carbapenem, colistin and doxycycline resistant *A. baumannii* during 2019–2020 in public hospitals of Lahore, Pakistan.

Specimens	Frequency based on site of infection	Frequency of MDR + carbapenem resistant isolates	Frequency of MDR + doxycycline/minocycline resistant isolates	Frequency of MDR + colistin resistant isolates
Sputum and tracheal wash	28 (19,4%)	24 (85,7%)	**8 (28,5%)**	**7 (25%)**
Blood and central venous pressure (CVP) tip	56 (38,8%)	50 (89,2%)	3 (5,3%)	2 (3.5%)
Wound and Pus	23 (16,3%)	19 (82,6%)	1 (4,3%)	0
Urine and urinary catheter	36 (25%)	33 (91,6%)	0	2 (5,5%)
Others	7	0	2 (28,5%)	0
Total	150	126 (84%)	14 (9,33%)	11 (7,3%)

**TABLE 2:** Molecular and drug resistant features of colistin resistant isolates.

Isolate ID	Specimen	Hospital	OXA51 typing	IsbA1	Drug susceptibility	Drug resistance
2201	Tracheal wash	SIMS	66	−	TOB	CL, IPM, AMC, LEV, CTX, FEP, TPZ, SXT, CAZ, MH, AK
2202	Tracheal wash	KE	66		DO, MH	CL, IPM, AMC, TOB, LEV, CTX, FEP, TPZ, SXT, CAZ, AK
2203	Sputum	Jinnah	66	−	TOB, DO, MH	CL, SXT, IPM, AMC, LEV, CTX, FEP, TPZ, CAZ, AK
2204	Tracheal wash	Jinnah	66	−	--	CL, IPM, AMC, TOB, LEV, CTX, FEP, TPZ, DO, SXT, CAZ, MH, AK
2205	Sputum	SIMS	66	+	TOB, DO, SXT, MH	CL, IPM, AMC, LEV, CTX, FEP, TZP, CAZ, AK
2206	Blood	KE	90	−	TPZ	CL, IPM, AMC, TOB, LEV, CTX, FEP, DO, SXT, CAZ, MH, AK.
2207	Tracheal wash	Jinnah	66	−	IPM, DO, AK	CL, AMC, TOB, LEV, CTX, FEP, DO, SXT, CAZ, MH.
2208	Tracheal wash	SIMS	66		--	CL, CAZ, IPM, AMC, TOB, LEV, CTX, FEP, TPZ, DO, SXT, MH, AK.
2209	Blood	Jinnah	33	−	TOB, DO, MH	CL, IPM, AMC, LEV, CTX, FEP, TZP, SXT, CAZ, AK.
2210	Urine	Jinnah	213	−	IPM, AMC, LEV, TZP	CL, AK, TOB, CTX, FEP, DO, SXT, CAZ, MH
AB-4/*Acinetobacter baumannii CR-4*	Urine	Ittefaq	33	+	DO, MH	CL, IPM, AMC, LEV, CTX, FEP, TZP, SXT, CAZ, AK.

KE, King Edvard medical university; SIMS, Services institute of medical sciences; SAM ampicillin/sulbactam, TZP, piperacillin/tazobactam, FEP, cefepime; CTX, cefotaxime; CAZ ceftazidime; CRO, ceftriaxone; IPM, imipenem; MEM, meropenem; CST, colistin; PMB, polymyxinB, DOX, doxycycline; MIN, minocycline; GM, gentamicin; AMK, amikacin; TOB, tobramycin; CIP, ciprofloxacin; LVX, levofloxacin; SXT, trimethoprim/sulfamethoxazole, CL, colistin.

### Identification of novel mutations associated with colistin resistance

In order to monitor the potential effect of colistin resistance on the fitness and virulence of *A. baumannii,* a colistin resistant derivative of the well characterized colistin sensitive strain *A. baumannii*17978 was obtained by challenging it with sub optimal concentration of 0,25 ug/ml colistin by passing the bacteria through three consecutive overnight subcultures. The resulting colistin resistant variant of *A. baumannii* 17978, hereafter referred to as CR-1, exhibited a MIC value of 16 ug/ml for colistin. Whole genome sequencing of the isolate CR-1 was performed to assess adaptive response to colistin at genetic level. Mutations in one of the genes involved in encoding LPS biosynthetic machinery *lpxA, lpxC* or *lpxD* or mutations in *pmrAB* operon involved in the addition of phospho ethanolamine on LPS have been known to cause colistin resistance. However, no mutation was detected in these genes in the CR-1 isolate (Whole genome sequence Accession no. JANIHK000000000). Hence, the colistin resistance acquired by CR-1 appeared independent of mutations in these genes. In depth analysis of the whole genome sequence of CR-1 identified four single base pair mutations in loci AIS_1585, AIS_2445, AIS_3424 and AIS_2454 in the genome of CR-1 (Table 3). None of these point mutations have been shown to be associated with colistin resistance previously. Interestingly, the transposons mutants of these four genes along with several other mutants were shown to be associated with a salt induced colistin tolerance phenotype (Hood et al., 2013) suggesting the relevance of these single nucleotide mutations in colistin resistance acquired by CR-1. These mutations appear to be associated with colistin resistance phenotype. However to find role of these mutations in colistin resistance further investigations are required.

**TABLE 3:** Mutations associated with colistin tolerance in *A. baumannii* 17978 as revealed from whole genome sequencing of CR-1.

Loci	Gene product	Mutation
*A1S_1585*	DNA helicase	GLu170Val
*A1S_2445*	ATP binding protein	Phe215Gln
*A1S_3424*	Lipo protein precursor Nlp	Leu119Phe
*A1S_2454*	L-24-diaminobutyrate:2 ketoglutarate 4-aminotransferase	Gly122Asp

### The reduced virulence associated with colistin resistance can be independent of mutations in *pmrAB, lpxA, lpxB,* or *lpxD*

**FIGURE 1:**
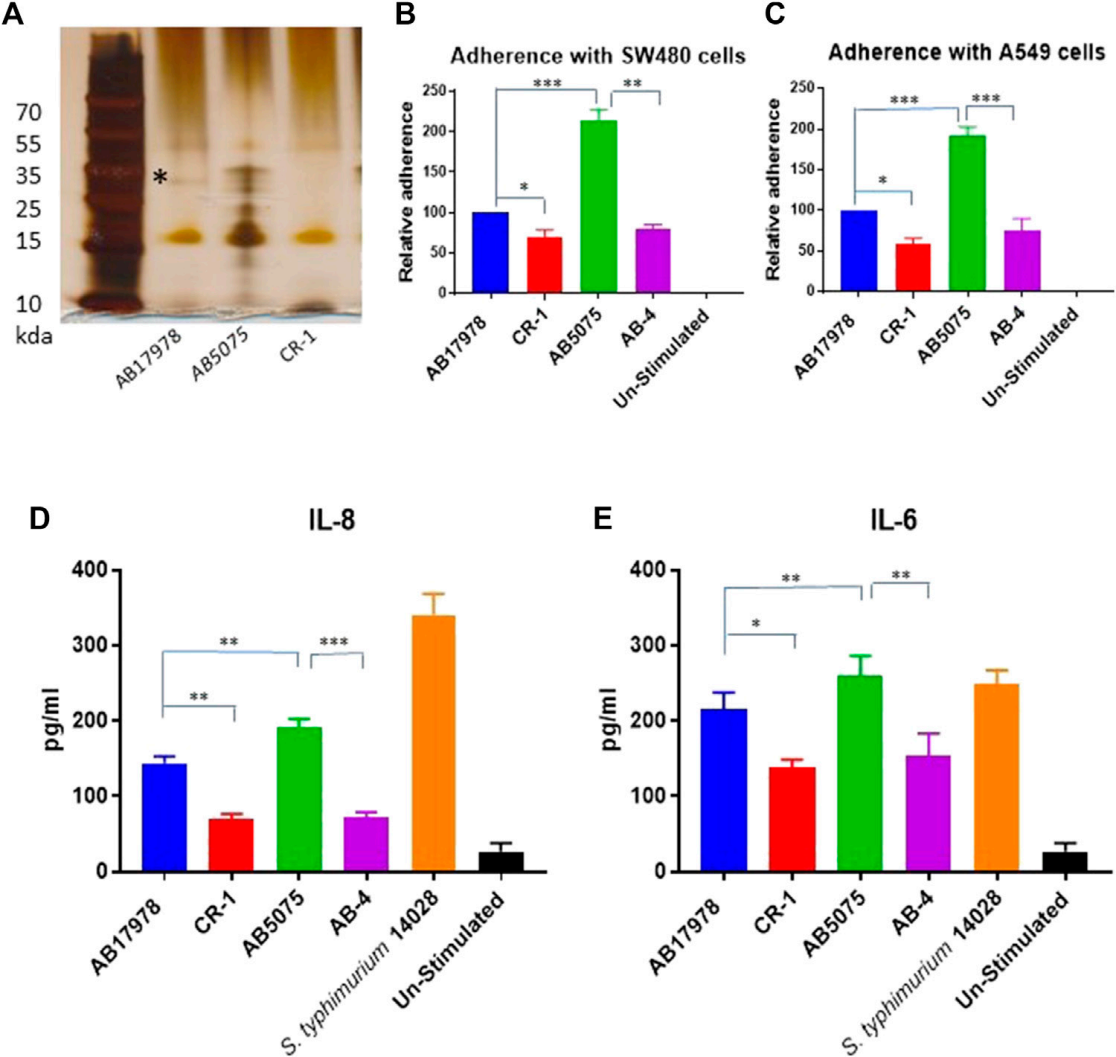
Consequences of colistin resistance in *A. baumannii* for capabilities to adhere with epithelial cells and to pro-inflammatory immune response induction. **(A)** Silver stained polyacrylamide gel elucidating a total LPS content of CR-1 as compared to the parental strain *A. baumannii* 17978 (AB 17978) and the hyper virulent strain AB5075. The band indicated by asterisk is corresponding to 35 kda with respect to protein ladder is diminished in CR-1 isolate **(B)** Relative adherence of *A. baumannii* strains with respect to *A. baumannii* 17978 wild type with SW480 cell lines and **(C)** A549 cell lines upon 4 h of co-infection with MOI of 1:100. Absolute values of cytokines IL-8 **(D)** and IL -6 **(E)** induced by bacterial strains from SW480 cells upon 4 h of co-infection with MOI of 1:100. *S. typhimurium* 14028 was used as a positive control. Un-stimulated refers to cells without any infection. Bars show mean ± standard deviation from at least five independent biological experiments performed in three technical replicates. Statistical significance is indicated by *p < 0.05, **p < 0.01 and ***p < 0.001 using non-parametric *t* test.

The CR-1 isolate was then tested for LPS biogenesis. Silver staining of LPS preparations resolved in acrylamide gel identified that CR-1 lacks a distinct band of lipopolysaccharide rather than an overall decrease in LPS content (Figure 1A). It is known that *A. baumannii* acquires colistin resistance at the cost of virulence loss due to mutations in *lpxA*, *lpxC*, *lpxD* or *pmrAB* (Da Silva and Domingues, 2017). However, these genes were intact in CR-1. Therefore, the CR-1 isolate was then tested for virulence phenotypes and capability to induce a pre-inflammatory immune response in epithelial cells. Lung epithelial cell line A549 and rectal carcinoma epithelial cells SW480 were infected with CR-1 and wild type *A. baumannii* 17978. The adherence capability of CR-1 with both cell lines was significantly decreased (Figures 1B,C). Consistently, the pro-inflammatory response induced by CR-1 in SW480 cells, measured as IL-6 and IL-8 levels, was significantly decreased in case of the CR-1 isolate as compare to wild type (Figures 1D,E). Since colistin resistance in CR-1 was acquired under laboratory conditions, to validate these findings, naturally colistin resistant clinical isolate AB-4 was also tested for interaction with epithelial cells in comparison to colistin sensitive hyper virulent strain *A. baumannii* AB5075*.* The cell adherence and cytokine induction capabilities of AB-4 were significantly decreased as compare to *A. baumannii* AB5075 (Figures 1B–D). The identity and molecular typing of AB-4 was determined by whole genome sequencing. The multi locus sequence (MLS) typing shows that the isolate belongs to international clone II and MLS type 2 (Supplementary Table S2). AB-4 is found to be equipped with beta lactamases OXA-66 and *blaADC25* of *A. baumannii*. The broad range beta lactamase encoding genes *bla-PER-1* and *blaOXA-23* were also detected in the genome of AB-4. In addition, streptogramin B and macrolide resistance genes *msr*(E) and *mph* (E) were detected. The isolate was found to harbour aminoglycoside resistance genes *aph* (3”)-Ib, *aph* (3”)-VIb, *aph* (3”)-Id, *aph* (3”)-Via, *aph* (3”)-Ia and *armA* and sulfonamide resistance gene *sul*-2 (Supplementary Table S3).

**FIGURE 2:**
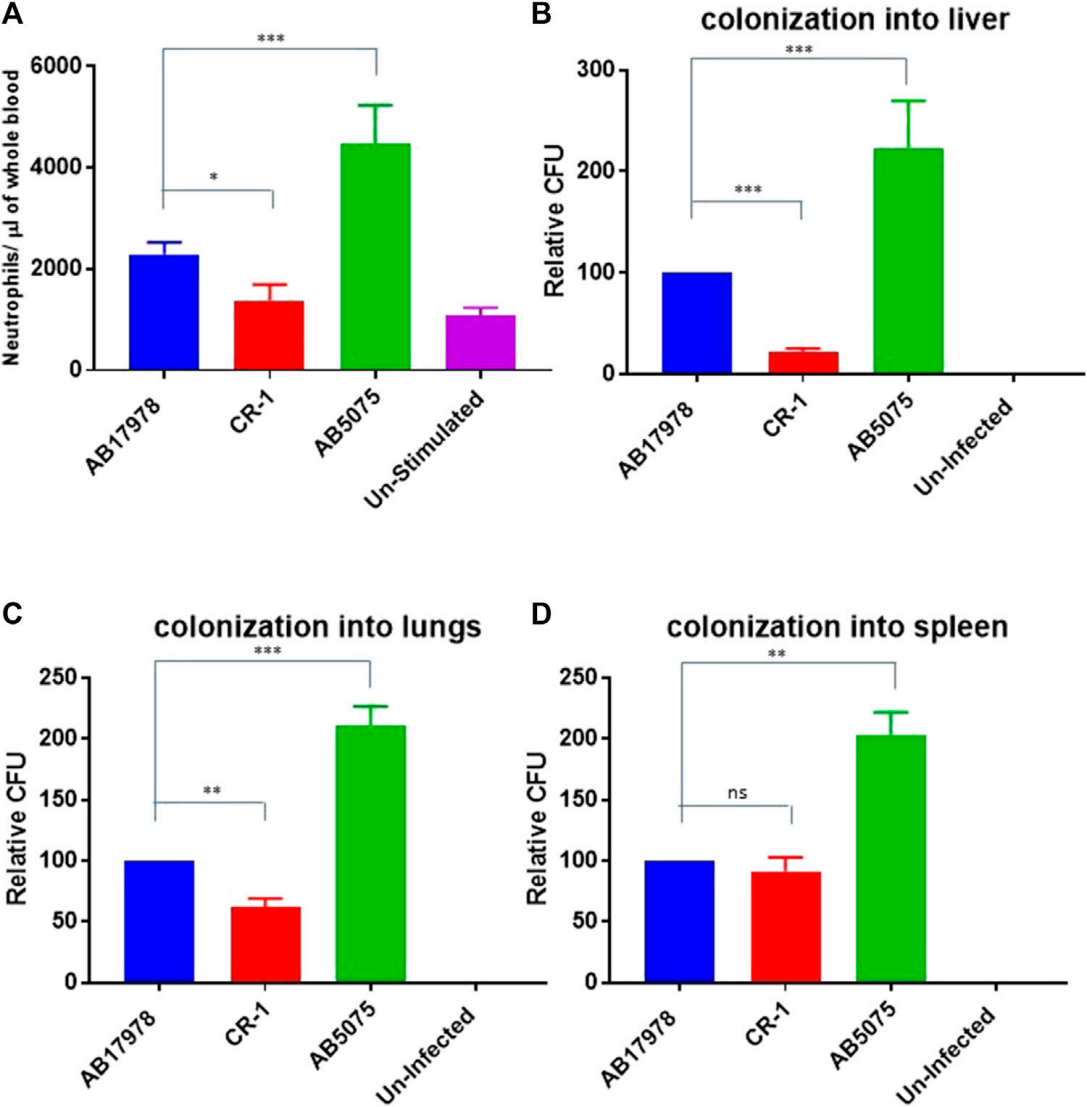
Consequences of colistin resistance in CR-1 for virulence in BALB/c mice. **(A)** Total neutrophil counts in the whole blood of mice withdrawn through heart puncture after 20 h of infection. Number of viable bacterial counts shown as colony forming unit (CFU) count into liver **(B)**, lungs **(C)** and spleen **(D)** of the mice sacrificed after 20 h of intraperitoneal innocula consisting of 10^7^ cells suspended in phosphate buffer saline. Bars show mean ± standard deviation of the individual values obtained from six mice in each group. Statistical significance is indicated by *p < 0.05, **p < 0.01and ***p < 0.001 using non-parametric *t* test.

These *in vitro* findings triggered us to test CR-1 for virulence potential and inflammatory response associated with colistin resistance in a mouse infection model. For that, BALB/c mice were intraperitoneally infected with wild type *A. baumannii* 17978 and its colistin resistant mutant strain CR-1. The hyper virulent strain AB5075 was used as a positive control to establish virulence phenotypes. The mice infected with *A. baumannii* AB5075 and *A. baumannii* 17978 exhibited a strong inflammatory response with respectively 4-fold and 2-fold increase in neutrophil count in the blood after 24 h of the post infection (Figure 2A). The colistin resistant mutant strain CR-1 also induced an IL-8 and neutrophil increase upon intraperitoneal infection. However, there was a significant decrease in the number of colonized bacteria into lungs and liver of the mice infected with colistin resistant isolates as compare to colistin sensitive isolates (Figures 2B,C). There was no significant difference detected in the colonization of CR-1 bacteria into spleen (Figure 2D). Altogether these findings suggest that the colistin resistant strain CR-1, although it harbours intact *lpxA* ^*+*^ *, lpxC* ^*+*^ *, lpxD* ^*+*^ and *pmrA* ^*+*^ *B* ^*+*^ loci although it exhibited a significant reduced virulence in the mouse model.

### Synergism of doxycycline and colistin in colistin resistant isolates

Nine out of 11 colistin resistant isolates were detected susceptible to tetracycline drugs, doxycycline and minocycline (Table 2). In order to determine potency of doxycycline against colistin resistant isolates, time dependent bactericidal and bacteriostatic effects of doxycycline in the presence and absence of colistin were investigated in a representative isolate. The colistin resistant but doxycycline susceptible strain AB-4 was selected as a representative strain for this purpose.

**FIGURE 3:**
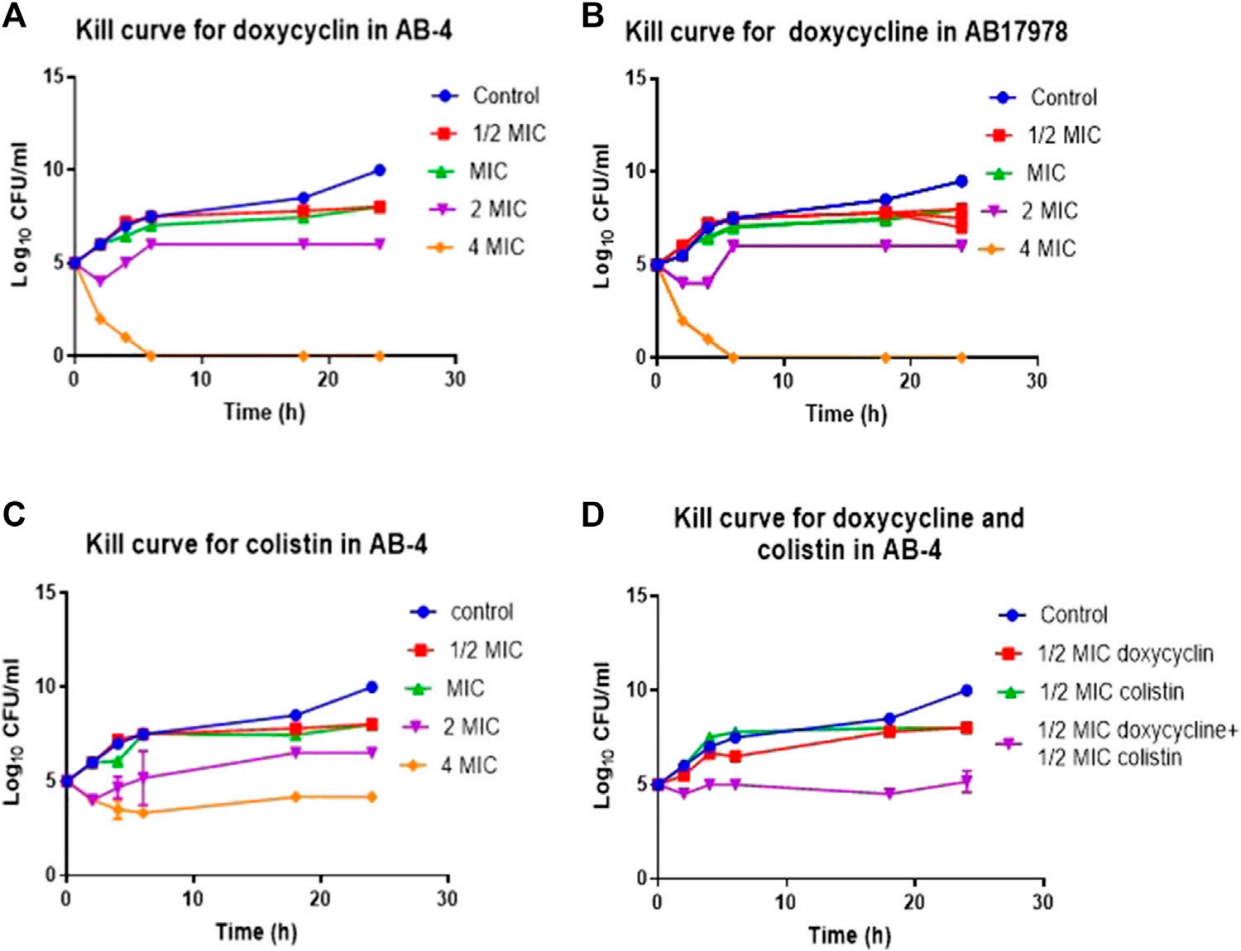
Time kill curve demonstrating *in vitro* efficacy of doxycycline in colistin resistant clinical isolate AB-4 **(A)** Kill curve of AB-4 upon the treatment with different concentrations of doxycycline. The MIC value for doxycycline in AB-4 is 0.5 ug/ml **(B)** Kill curve of *A. baumannii* 17978 (AB17978 upon the treatment with different concentrations of doxycycline. The MIC value for doxycycline in AB17978 is 0.125 ug/ml **(C)** Kill curve of AB-4 upon the treatment with different concentrations of colistin. The MIC value for colistin in AB-4 is 16 ug/ml **(D)** Kill curve of AB-4 upon the treatment with colistin and doxycycline. Bars show mean ± standard deviation from at least five independent biological experiments.

In time-kill curve studies, doxycycline showed a 4-log_10_ reduction in CFU/ml at 4xMIC concentration in comparison to growth control after 2 h of incubation and 3-log_10_ reduction at 2xMIC after 18 h of incubation against AB-04 and AB17978 (Figures 3A,B). There were no viable bacteria detected with the treatment of 4xMIC after 8 h of incubation. However, there was 4-log_10_ reduction of CFU with 2xMIC treatment and 2-log_10_ reduction with MIC with a ½ MIC treatment of doxycycline.

The MIC for colistin of AB-4 was 16 μg/ml. In the time-kill curve studies, colistin showed a 2-log_10_ reduction in CFU/ml at 4xMIC concentration in comparison to growth control after 2 h of incubation and a 1-log_10_ reduction at 2xMIC after 18 h of incubation (Figure 3C). Colistin showed bactericidal effect towards strain AB-4 at a concentration twice of MIC at second hour of incubation. A bactericidal effect was also noted at the second hour of incubation at a concentration of four times of their MICs whereas no bactericidal effect was found at their MIC during 24 h of incubation.

Subsequently, the time kill assay was performed to assess the effect of colistin in the presence of doxycycline with strain AB-4 at ½ MICs of colistin and doxycycline (Figure 3D). No bactericidal effect was observed with ½ MICs of doxycycline or colistin alone in first hour of incubation. However, treatment with both doxycycline and colistin at ½ MICs each significantly reduced the CFU, 3-5 log_10_ at all tested time points. This finding suggests that a synergetic effect of antimicrobial activity of colistin and doxycycline exists in colistin resistant isolate AB-4.

### Efficacy of an extract from the herb *Saussurea lappa* against colistin resistant *A. baumannii* isolates

**FIGURE 4:**
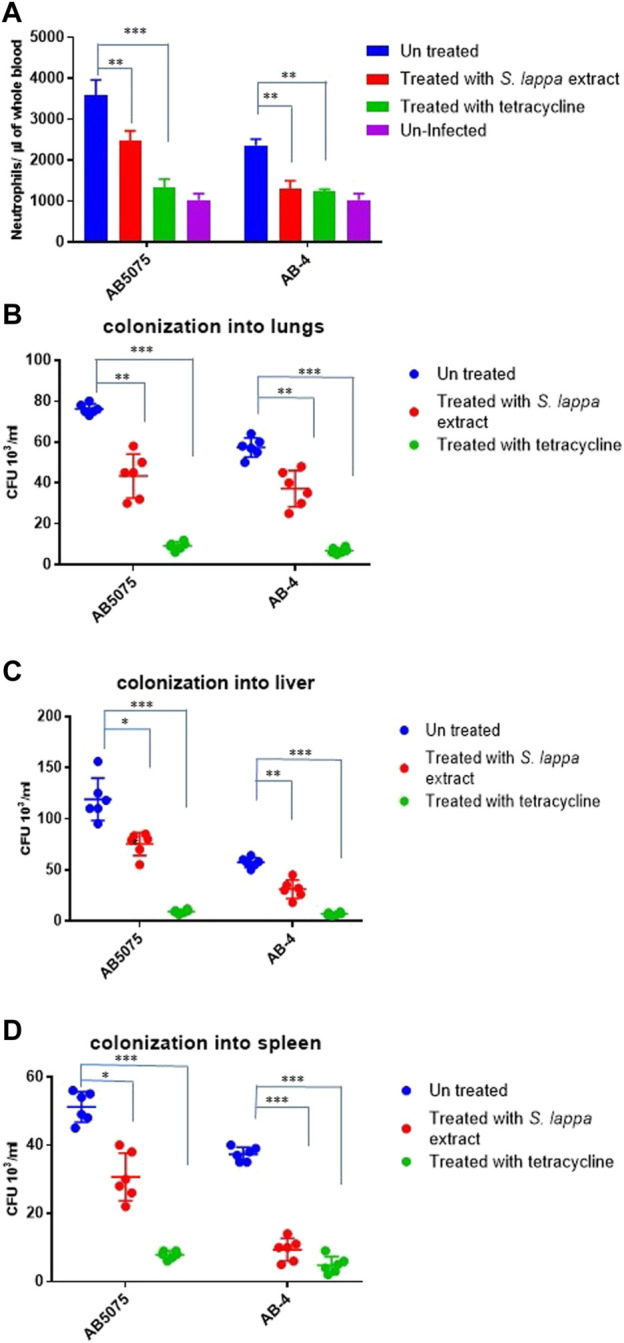
Effect of the oral administration of the extract from *S. lappa* in mice infected with the AB5075 or AB-4 strains of *A. baumannii* **(A)** Total neutrophil counts in the whole blood of mice withdrawn through heart puncture after 20 h of infection. Number of viable bacterial counts shown as colony forming unit (CFU) count in liver **(B)**, lungs **(C)** and spleen **(D)** of the mice sacrificed after 20 h of intraperitoneal infection consisting of 10^7^ cells suspended in phosphate buffer saline. Bars show mean ± standard deviation of the individual values obtained from six mice in each group. Statistical significance is indicated by *p < 0.05 and **p < 0.01 and ***p < 0.001 using non-parametric *t* test.

Considering very limited, treatment options against colistin resistant isolates (Table 1), we were interested to explore other alternative treatment strategies. In this regard, extracts of the traditional medicinal plant *Saussurea lappa* have been shown to possess antimicrobial activity against Gram-negative and Gram-positive and anti-inflammatory activity in tracheal inflammation (Hasson et al., 2013; Rao Vadaparthi et al., 2015; Pyun et al., 2018). *In vitro* antimicrobial activity of various fractions of the herb *Saussurea lappa* was tested against colistin resistant *A. baumannii* isolates*.* However, a remarkable antimicrobial activity of *S. lappa* against these isolates was not observed *in vitro* at the concentration of less than up to the concentration of 10 mg/ml. In order to assess its anti-inflammatory role, a water extract of *S. lappa* was tested in the mouse model of systemic infection against the extreme drug resistant isolate *A. bauamnnii AB-4* upon intraperitoneal infection and with the hyper virulent strain *A. baumannii* AB5075. The mice were treated with the water-soluble fraction from *S. lappa*. For that, a dose consisting of 10 mg of *S. lappa* powder was orally administrated to each mouse after 3 h of intraperitoneal infection. The inflammation induced by *A. baumannii* strains was measured as IL-8 levels and total neutrophil count in the blood of infected mice after 20 h of infection. There was a statistically significant reduction in IL-8 levels and total neutrophil count in the blood of the infected mice treated with *S. lappa* or tetracycline as compare to uninfected group (Figure 4A). This finding suggests that the compound from *S. lappa* modulates the inflammatory response induced by *A. baumannii* infection. Also, there was a significant decrease in numbers of viable bacteria colonized into liver and lungs of infected mice treated with *S. lappa* extract or tetracycline as compare to the untreated group (Figures 4B–D). Altogether, these findings suggest that the water-soluble fraction of *S. lappa* possess some immunomodulatory compound that may be used to treat *A. baumannii* infection in mice.

### Immunomodulatory effect of *Saussura lappa* with respect to colony phase variation in *Acinetobacter baumannii* AB5075

**FIGURE 5:**
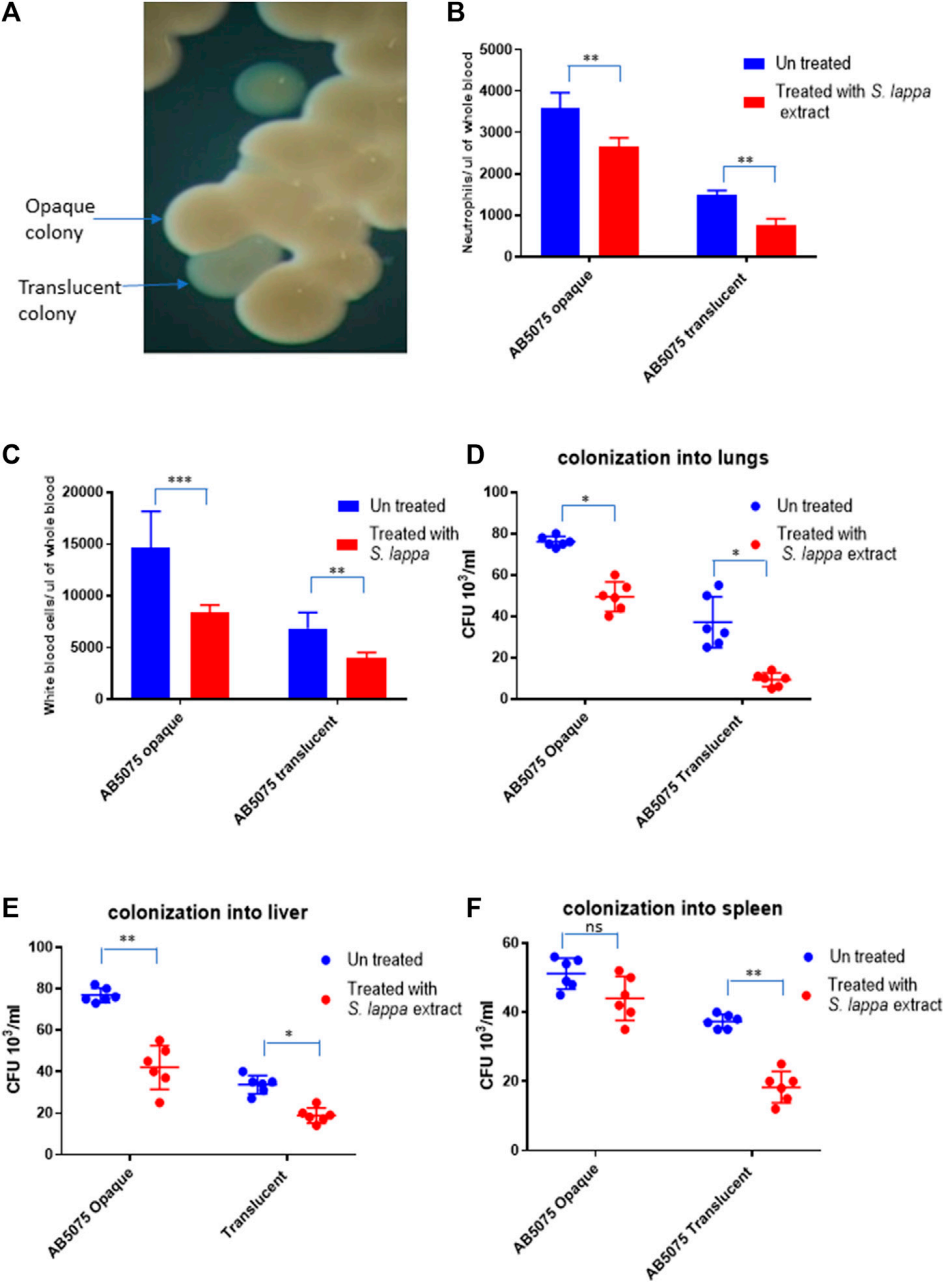
Effect of the extract from *S. lappa* in mice infected with opaque and translucent colony variants of *A. baumannii* AB 5075 **(A)** Stereomicroscopic image of opaque and translucent colonies in a typical LB agar culture of *A. baumannii* AB 5075. **(B)** Total neutrophils count in the whole blood of mice withdrawn through heart puncture after 20 h of infection. **(C)** Total white blood cells counts in the whole blood of mice withdrawn through heart puncture after 20 h of infection **(D)** Number of viable bacterial counts shown as colony forming unit (CFU) count in the lungs, liver **(E)** and spleen **(F)** of the mice sacrificed after 20 h of intraperitoneal innocula consisting of 10^7^ cells suspended in phosphate buffer saline. Bars show mean ± standard deviation of the individual values obtained from six mice in each group. Statistical significance is indicated by *p < 0.05, **p < 0.01 and ***p < 0.001 non-parametric *t* test.

*A. baumannii* AB5075 displays a recognized capability of switching into an avirulent subpopulation of bacterial cells that appears as translucent colonies on agar plates (Figure 5A) (Tipton et al., 2015; Chin et al., 2018; Ahmad et al., 2019). The findings of the immunomodulatory role of *Saussurea lappa* extracts towards *A. baumanii* infection inspired us to investigate whether such effects would be limited to a certain subpopulation of *A. baumannii* AB5075. The effect of the *S. lappa* extract was therefore tested upon infecting mice with opaque and translucent colony variants of *A. baumannii* AB5075*.* As reported previously, the opaque variant was more virulent as compare to translucent counterpart in the mouse. The administration of *S. lappa* extract to mice groups infected with opaque and translucent variants exerted similar effect in lowering cytokine levels, white blood cell count neutrophils and colonization of bacteria into organs (Figures 5B–E).

## Discussion

Colistin is a last resort drug for the treatment of carbapenem resistant *A. baumannii* infections. The finding on the emergence of colistin resistance among several clonal types of carbapenem resistant *A. baumannii* isolates in Pakistani hospitals is an alarming situation. Particularly, there were two isolates detected with resistance to all tested antibiotics from two different hospitals. Also, the emergence of colistin resistance among carbapenem resistant isolates have been reported in other parts of the world (Abdulzahra et al., 2018; Al-Kadmy et al., 2019; Thadtapong et al., 2021). If the situation is not tackled vigilantly, the frequent outbreaks of pan-drug resistant strains of *A. baumannii* could be expected in the near future.

Colistin resistant clinical isolate AB-4 as well as colistin resistant derivative of reference strain AB17978CR-1 appeared deficient in their capabilities to induce a proinflammatory response and virulence in mice. It is a well evident fact that *A. baumannii* acquires colistin resistance at the cost of virulence potential (Da Silva and Domingues, 2017). *A. baumannii* can acquire resistance to colistin via complete loss of lipopolysaccharide (LPS) biosynthesis due to mutations in the *lpxA*, *lpxC* and *lpxD* genes (Moffatt et al., 2010a). Consistently, a LPS-deficient derivative of the ATCC 19606 strain was not only defective in causing virulence in mice but also acquired growth defects (Carretero-Ledesma et al., 2018). LPS loss altered several other bacterial traits such as biofilm formation, motility and growth rate in the presence of disinfectants. The mutations in genes encoding the LPS biosynthesis machinery were found to be associated with higher fitness costs than in case of mutations in the *pmrAB* operon (Mu et al., 2016). Similarly, colistin resistance associated with mutations in *pmrB* upon the long-term exposure of colistin with extreme drug resistant isolates did not cause any virulence defect (Durante-Mangoni et al., 2015). In contrast, LPS loss mediated due to mutations in the *lpx* operon and phospho ethanolamine addition mediated by mutations in *pmrAB,* both caused a defect in fitness of *A. baumannii* 19606 while *pmrAB* mutants acquired a higher viability loss as compared to *lpx* mutants upon interaction with A549 epithelial cells. Also, host dependent effects of virulence loss was observed in *lpx* and *pmrB* mutants in *C. elegans* and mice models (Beceiro et al., 2014).

The findings suggest that the cause of colistin resistance determines the virulence potential of *A. baumannii*. The treatment strategy could also have differential impact on colistin resistance with or without alteration in LPS biosynthesis. Therefore, a detailed investigation is required to determine the impact of second line drugs such as doxycycline, minocycline and doxycycline on treatment of colistin resistant *A. baumannii.* Doxycycline is found to be an effective choice to treat colistin resistant *A. baumannii*. Particularly, in the presence of colistin ½ MIC (8 ug/ml), the bactericidal effect of doxycycline is found to increase at least 3-log. A systematic evaluation of alternative antibiotics against colistin resistant *A. baumannii* will be required.

Attempts to find alternative treatment options for colistin resistant *A. baumannii* lead us to identify the root extract of *Saussurea lappa* as an effective immunomodulatory agent to treat such infections. However, *in vitro* antimicrobial activity was not detected against colistin resistant *A. baumannii.* In contrast, the root extract of *S. lappa* has been shown to possess *in vitro* antibacterial activity against several types of Gram-positive and Gram-negative bacteria such as *Staphylococcus aureus*, *Pseudomonas aeruginosa*, *Escherichia coli*, *Klebsiella pneumoniae*, and carbapenem resistant *A. baumannii* (Hasson et al., 2013).

The idea to use plant extracts as anti-bacterial agent is not new but have been tested in several plants successfully. For example, the extracts of *Lythrum salicaria* was shown to possess antimicrobial activity against *A. baumannii* and *P. aeruginosa* (Ertugrul Guclu et al., 2013). Similarly, herbs like *Syzygium aromaticum* and *Cinnamomum zeylanicum,* have been shown to have promising results against multidrug-resistant *A. baumannii* (Burt, 2004). The use of epigallocatechin gallate along with curcumin harbours a synergetic effect to inhibit the growth of *A. baumannii*. The combinational use of both herbs at the concentration of 4 µg/ml inhibits the growth of *A. baumannii* without any antagonistic effect (Betts and Wareham, 2014). The extracts of plant *Magnolia dealbata* have also been shown to exhibit anti-bacterial activity against many bacteria such as *P. aeruginosa, Clavibacter michiganensis, A. baumannii and A. lwoffii.* Honokiol and magnolol are important active ingredients in this plant that possess antimicrobial activity (Jacobo-Salcedo Mdel et al., 2011).

Plant extracts are not only found to exhibit antimicrobial activity but there are multiple plant derived molecules found to enhance antimicrobial potential of existing antibiotics. For example, plant derived compounds tannic acid and ellagic acid have been shown to enhance antimicrobial activity of many drugs such as novobiocin, chlorobiocin, coumermycin rifampicin and fusidic acid (Chusri et al., 2009). An alkaloid compound, berberine, found in different kinds of plants such as *Rhizoma coptidis, Berberis fremontii* and *Hydrastis canadensis* possess antibacterial activities (Lewis and Ausubel, 2006). Plant derived antibacterial molecules are generally weak but work better in synergy with antibiotics (Lewis and Ausubel, 2006). Picatechin, a tea polyphenol having no antibacterial properties can potentiate theaflavin *in vivo* and thus increases its activity against *A. baumannii* and *Stenotrophomonas maltophilia* isolates. The probable mechanism may be that epicatechin inhibits theaflavin oxidation thus enhancing its antibacterial effect, but the exact mechanism of synergy is not yet understood and needs further study (Betts and Wareham, 2014).

Water extracts of the roots of *S. lappa,* although it lacks an antimicrobial activity *in vitro* against *A. baumannii,* showed a significant potential of suppressing virulence and inflammation induced upon the infection by *A. baumannii* in the mouse model. The key ingredients of this herb include sesquiterpene lactones and dehydrocostus lactone (Rao Vadaparthi et al., 2015). The immune modulatory role of sesquiterpens in the inhibition of proinflammatory cytokine production and lymphocyte proliferation is well evident (Koch et al., 2001). Also, Dehydrocostus lactone has been shown to suppresses allergic airway inflammation by binding to dimerized translationally controlled tumor protein in a mouse model and thus possesses important role in immunomodulation (Pyun et al., 2018). Further studies are required to reveal the active ingredient of *S. lappa* involved in the presently observed modulation of immune response that suppress the systemic infection of *A. baumannii* in mouse.

In summary, we have elucidated the status of colistin resistance in carbapenem resistant *A. baumannii* isolates from Pakistan, the synergetic effect of colistin and doxycycline against colistin resistant isolates and the inhibitory potential of an extract from *S. lappa* on the infection and inflammation induced by *A. baumannii* in mice. However, further detailed analysis is required to identify the active ingredient(s) and mechanisms of this inhibitory role.

## Materials and methods

### Bacterial strains

Clinical isolates of *A. baumannii* (*n* = 150) were collected from clinical samples of patients visited or hospitalized in Jinnah hospital, Ittefaq Hospital Lahore, Services Institute of Medical Sciences, King Edward Medical University Laboratory and Mayo Hospital Lahore during 2019–2020. The strains were identified by their morphological and biochemical characteristics using API 20-NE (Bio Merieux, France) following manufacturer`s instructions. *A. baumannii*s 17978 was used for isolation of a colistin resistant derivative CR-1. The hyper virulent strain AB5075 and its opaque and translucent colony variants were used for infection tests (Ahmad et al., 2019). *Escherichia coli* ATCC 25922 was used as a control strain in MIC value tests. *Salmonella typhimurium* 14028 was used as positive control to induce proinflammatory response from epithelial cells. The strains were stored at minus 80°C in 10% glycerol prepared in LB broth.

### Antimicrobial susceptibility testing

Antimicrobial susceptibility testing was performed by the standard Kirby-Bauer disk diffusion method using cation adjusted Mueller-Hinton agar (MHA) (Oxoid, United Kingdom), according to Clinical Laboratory Standards Institute (CLSI) guidelines (Clinical and Laboratory Standards Institute (CLSI), 2019) Antibiotic discs (Oxoid, United Kingdom) used were piperacillin (100 µg), ampicillin-sulbactam (10 µg/10 µg), piperacillin-tazobactam (100 µg/10 µg), ticarcillin-clavulanic acid (75µ/10 µg), ceftazidime (30 µg), ceftriaxone (30 µg), cefepime (30 µg), imipenem (10 µg), gentamicin (10 µg), doxycycline (30 µg) and ciprofloxacin (5 µg).

### MIC determination of colistin and tetracycline

The minimal inhibitory concentration of colistin was determined by micro broth dilution method. The base material colistin (Glaxosmith Kline pharmaceuticals) was prepared in water and tetracycline in 70% ethanol and stored at minus 20°C. MICs of all strains for each antibiotic were determined by standard microbroth dilution method. Bacterial inoculum equivalent to 0.5 McFarland (5 × 10^8^) was prepared and diluted 1:10 to achieve the final inoculum of 5 × 10^7^ CFU/ml. Concentration range of the antibiotics to be tested was; 0.125–256 µg in MHA using 96 well plates. The plates were incubated for 24 h at 37°C and the lowest concentration at which bacterial growth was completely inhibited was noted and declared as MIC. *Escherichia coli* ATCC 25922 was used as a control strain. MIC results were read and interpreted according to the CLSI 2019 breakpoints for *A. baumannii*.

### Strain typing

The colistin resistant isolates were typed using single-locus molecular schemes based on the allelic identity of the *A. baumannii*-intrinsic *bla*_OXA-51-like_ gene (Pournaras et al., 2014). PCR amplification of *bla*_OXA-51-like_ was performed using in-house designed primers (Supplementary Table S1) and followed by Sanger sequencing of the amplicons. The thermal cycling program used for PCR consisted of: initial denaturation; 94°C for 5 min: 30 cycles of amplification: denaturation at 95°C for 20 s; annealing at 56°C for 20 s, and extension at 72°C for 1 min and 30 s: Final extension; 72°C for 8 min. This approach was able to detect the occurrence of insertion sequence (IS) elements, such as IS*Aba1*, in the bordering regions of *bla*_OXA-51-like_.

### Whole-genome sequence analysis

The whole genome sequencing of colistin resistant clinical isolate AB-4 and colistin resistant variant of *A. baumannii* 17978 CR-1 was performed using the MiSeq Desktop Sequencer and MiSeq Reagent Kit v3 (Illumina, San Diego, CA, United States). DNA preparation, library construction, and genome sequencing were done according to the manufacturer’s instructions. Sequence data were assembled and analyzed using the CLC genomics workbench (v7.0.4; CLC bio, Aarhus, Denmark). The whole genome sequence of *A. baumannii* ATCC 17978 (DDBJ/EMBL/GenBank database accession number CP000521) was used as a reference sequence.

The MLST web-based search engine, hosted by the Center for Genomic Epidemiology in Denmark (http://www.genomicepidemiology.org/), was used to assign the isolate into STs according to the Institute Pasteur’s MLST scheme (http://www.pasteur.fr/mlst) (Larsen et al., 2012). The occurrence of acquired antimicrobial resistance genes was detected using the Res Finder service, also hosted by the Center for Genomic Epidemiology in Denmark [24]. The occurrence of resistance genes was verified, and genetic surroundings were annotated based on the yields of nucleotide similarities obtained using the Basic Local Alignment Search Tool (http://blast.ncbi.nlm.nih.gov/Blast.cgi) against the “Nucleotide collection (nr/nt)” and/or “Whole-genome shotgun contigs (wgs)” databases (Zhang et al., 2000).

### Nucleotide sequence accession numbers

Draft genome sequences of the isolates were deposited in the DDBJ/EMBL/GenBank database under the BioProject accession number: PRJNA862891. The isolates described in this paper are AB-4 with accession number JANIEU000000000 and CR-1 with accession number JANIHK000000000.

### Time-kill assay

The colistin resistant strain *A. baumannii* AB-4 and colistin sensitive strain *A. baumannii* 17978 were selected for time kill assay. Bacterial suspensions equivalent to 1.0 MF (3 × 10^8^ CFU/ml) was prepared for each isolate to be tested by inoculating four to 5 colonies into 5 ml of CAMHB and incubating for 6 h. One ml of 1.0 MF suspension was diluted in 4 ml of sterile saline to achieve 6 × 10^7^ CFU/ml (1:5 dilution). The same was further diluted (1:100) by dispensing 0.1 ml of 1:5 diluted suspension in 10 ml of saline. The colony count was 6 × 10^5^ CFU/ml. This was used as a starting inoculum in this method.

MICs were determined by micro broth dilution method found to be 0,125 ug/ml in AB17978 and 0,5 ug/ml in AB-4. The following concentrations were tested along with growth and sterility control tubes; MIC, two times the MIC, four times the MIC, and one half the MIC.

The antibiotic agent was dispensed in a sterile screw capped glass tube containing 9.9 ml of CAMHB as 0.1 ml of 100-fold concentrated antibiotic agent. The tubes were incubated at 37°C, and test samples were taken at 0, 2, 4, 6, 18, 24 h after inoculation, for colony count. Serial dilution of contents were prepared and 0.1 ml aliquot of each dilution was plated on Tryptic Soya agar and incubated overnight at 37°C. Antibiotic concentration was determined that resulted in 3-log_10_ reduction in CFU/ml as compared to the growth control curve. The time was determined that was required to achieve 3-log_10_ reduction.

### Cell adherence assays

Lung epithelial cell line A549 and human rectal carcinoma epithelial cell line SW480 were tested for interaction with *A. baumannii*. Cells were cultured to confluence in 24-well cell culture treated plates in RPMI 1640 cell culture medium supplemented with fetal bovine serum at 37°C with a continuous supply of 5% CO_2_. After a change of the medium, confluent layers cells grown in wells of 24 well plates were infected with respective bacterial strains at MOI of 100. For cell infection experiments, bacterial strains were grown to the logarithmic growth phase in LB broth at 37°C. Upon infection, the plate was incubated at 37°C with 5% CO_2_ for 4 h. The eukaryotic cell monolayer was washed three times with PBS and subsequently to be treated with 1 ml of 5% trypsin. After dissociation of cells attached to the surface, harvested sample was diluted in PBS. The 2^nd^ dilution of every well was processed for enumeration of CFU by point inoculation following overnight incubation on LB agar plates. The CFU numbers of each strain were recorded. Bacterial adherence was determined as the percentage of bacterial cells associated with eukaryotic cells relative to the original inoculums. Each experiment was performed as five biological replicates and three technical replicates.

### Levels of IL-6 and IL-8 measurement

A-549 and SW-480 cells were cultured in 24-well plates in RPMI-1640 medium supplemented with 5% fetal bovine serum (FBS) at 37°C with a continuous supply of 5% CO_2_. After a change of the medium, confluent layers cells were infected with respective bacterial strains at MOI of 100. For cell infection experiments, bacterial strains were grown into the logarithmic growth phase in LB broth at 37°C. Then the bacteria were harvested by centrifugation at 5000 rpm for 10 min and resuspended in RPMI. After infection, cells were incubated at 37°C with 5% CO_2_ for 4 h. The suspension was, centrifuged after 4 h. The supernatant was analysed for production of IL-8 and IL-6 using ELISA according to manufacturer’s instructions (Bio Assay Technology Laboratory). *Salmonella typhimurium* 14028 was used a positive control to induce IL-8 from epithelial cells.

### Preparation of *Saussurea lappa* fractions for *in vitro* and *in vivo* testing against *A. baumannii*

The root of *S. lappa* weighing 200 g was washed with water and kept in incubator at 37°C until get dried. Upon drying, roots were crushed into powder form and dissolved in autoclaved distilled water. The mixture was incubated overnight at room temperature followed by gentle centrifugation. The supernatant was collected in sterile tubes. The contents were dried in rotary evaporator. Left over powder was dissolved in water at the concentration of 100 mg/ml. The MIC of this water extract was tested against colistin resistant isolates described in Table 2 using the method described above for colistin.

### Infection in mouse model

BALB/c mice were used in the study. All animals were maintained and treated in accordance with the recommended rules of the WMA Helsinki declaration and the permit granted by University of Health Sciences, Lahore, Pakistan, ethical and research committee wide letter number UHS/REG-19/ERC/1236.

For infection experiments, bacterial strains were grown on LB agar plats overnight at 37°C. From the overnight culture, the isolates were sub-cultured into LB broth until the logarithmic phase. An inoculum consisting of 10^7^ cells of the fresh culture in log phase were used to infect animals. Adult mice were inoculated with *A. baumannii* isolates by intraperitoneal injection. When required, animals were feed with 10 mg of *S. lappa* extract or 100 ug of tetracycline drug. Animals were maintained under standard laboratory conditions with free access to food and water throughout the experimental period. Mice were sacrificed 20 h post inoculation (hpi) after anesthesia. For neutrophil count, 500 micro liters of the blood were withdrawn through a heart puncture and stored in vials containing 50 ul of 0.5 M EDTA. The relevant organs (such as lungs, liver and spleens were aseptically removed and used for quantitative bacteriology. Organs were homogenized in sterile saline using aerosol-proof homogenizers. Aliquots (100 μl) of 10-fold serial dilutions of the homogenates were cultured on LB agar plates to quantify the number of viable *A. baumannii* organisms in the respective organs. Total white blood cells and neutrophil count was measured by hematology analzer (Beckman Coulter DxH 900) and cytokine levels were measured by ELISA according to manufacturer’s instructions (Bio Assay Technology Laboratory).

### Statistical analysis

Graph Pad Prism seven was used to construct graphs and perform statistical analysis of results. The type of statistical analysis preformed on each set of data is indicated in the respective figure legend.

## Data Availability

The datasets presented in this study can be found in online repositories. The names of the repository/repositories and accession number(s) can be found in the article/Supplementary Material.

## References

[B1] Abdulzahra A. T. Khalil M. A. F. Elkhatib W. F. (2018). First report of colistin resistance among carbapenem-resistant Acinetobacter baumannii isolates recovered from hospitalized patients in Egypt. New Microbes New Infect. 26, 53–58. 10.1016/j.nmni.2018.08.007 30224972 PMC6138847

[B2] Ahmad I. Karah N. Nadeem A. Wai S. N. Uhlin B. E. (2019). Analysis of colony phase variation switch in Acinetobacter baumannii clinical isolates. PLoS One 14, e0210082. 10.1371/journal.pone.0210082 30608966 PMC6319719

[B3] Al-Kadmy I. M. S. Ibrahim S. A. Al-Saryi N. Aziz S. N. Besinis A. Hetta H. F. (2019). Prevalence of genes involved in colistin resistance in acinetobacter baumannii: First report from Iraq. Microb. Drug Resist. 26, 616–622. 10.1089/mdr.2019.0243 31816255

[B4] Antunes L. C. Visca P. Towner K. J. (2014). Acinetobacter baumannii: evolution of a global pathogen. Pathog. Dis. 71, 292–301. 10.1111/2049-632X.12125 24376225

[B5] Ayoub Moubareck C. Hammoudi Halat D. (2020)., 9. Antibiotics (Basel), E119. 10.3390/antibiotics9030119 Insights into acinetobacter baumannii: A review of microbiological, virulence, and resistance traits in a threatening nosocomial pathogen Antibiotics 32178356 PMC7148516

[B6] Beceiro A. Moreno A. Fernández N. Vallejo J. A. Aranda J. Adler B. (2014). Biological cost of different mechanisms of colistin resistance and their impact on virulence in acinetobacter baumannii. Antimicrob. Agents Chemother. 58, 518–526. 10.1128/AAC.01597-13 24189257 PMC3910726

[B7] Begum S. Hasan F. Hussain S. Ali Shah A. (2013). Prevalence of multi drug resistant Acinetobacter baumannii in the clinical samples from Tertiary Care Hospital in Islamabad, Pakistan. Pak. J. Med. Sci. 29, 1253–1258. 10.12669/pjms.295.3695 24353731 PMC3858913

[B8] Betts J. W. Wareham D. W. (2014). *In vitro* activity of curcumin in combination with epigallocatechin gallate (EGCG) versus multidrug-resistant Acinetobacter baumannii. BMC Microbiol. 14, 172. 10.1186/1471-2180-14-172 24969489 PMC4083870

[B9] Burt S. (2004). Essential oils: their antibacterial properties and potential applications in foods--a review. Int. J. Food Microbiol. 94, 223–253. 10.1016/j.ijfoodmicro.2004.03.022 15246235

[B10] Carretero-Ledesma M. García-Quintanilla M. Martín-Peña R. Pulido M. R. Pachón J. Mcconnell M. J. (2018). Phenotypic changes associated with Colistin resistance due to Lipopolysaccharide loss in Acinetobacter baumannii. Virulence 9, 930–942. 10.1080/21505594.2018.1460187 29638177 PMC5955468

[B11] Chin C. Y. Tipton K. A. Farokhyfar M. Burd E. M. Weiss D. S. Rather P. N. (2018). A high-frequency phenotypic switch links bacterial virulence and environmental survival in Acinetobacter baumannii. Nat. Microbiol. 3, 563–569. 10.1038/s41564-018-0151-5 29693659 PMC5921939

[B12] Chusri S. Villanueva I. Voravuthikunchai S. P. Davies J. (2009). Enhancing antibiotic activity: a strategy to control acinetobacter infections. J. Antimicrob. Chemother. 64, 1203–1211. 10.1093/jac/dkp381 19861335

[B13] Coetzee J. Corcoran C. Prentice E. Moodley M. Mendelson M. Poirel L. (2016). Clinical alert-emergence of plasmid-mediated colistin resistance (MCR-1) among *Escherichia coli* isolated from South African patients: in practice. S. Afr. Med. J. 106, 449–450. 10.7196/samj.2016.v106i5.10710 27138657

[B44] Clinical and Laboratory Standards Institute (CLSI) (2019). Performance standards for antimicrobial susceptibility testing 29th edn. Wayne: Clinical and Laboratory Standards Institute (CLSI).

[B14] Da Silva G. J. Domingues S. (2017). Interplay between colistin resistance, virulence and fitness in acinetobacter baumannii. Antibiot. (Basel) 6, 28. 10.3390/antibiotics6040028 PMC5745471 29160808

[B15] Durante-Mangoni E. Del Franco M. Andini R. Bernardo M. Giannouli M. Zarrilli R. (2015). Emergence of colistin resistance without loss of fitness and virulence after prolonged colistin administration in a patient with extensively drug-resistant Acinetobacter baumannii. Diagn. Microbiol. Infect. Dis. 82, 222–226. 10.1016/j.diagmicrobio.2015.03.013 25858028

[B16] Ertugrul Guclu H. G. Mustafa Zengin Oguz Karabay (2013). Antibacterial activity of Lythrum salicaria against multidrug-resistant acinetobacter baumannii and *Pseudomonas aeruginosa* . Annu. Res. Rev. Biol. 4, 1099–1105. 10.9734/arrb/2014/7357

[B17] Evans B. A. Hamouda A. Abbasi S. A. Khan F. A. Amyes S. G. (2011). High prevalence of unrelated multidrug-resistant Acinetobacter baumannii isolates in Pakistani military hospitals. Int. J. Antimicrob. Agents 37 (6), 580–581. 10.1016/j.ijantimicag.2011.01.023 21481570

[B18] Gallardo-Godoy A. Muldoon C. Becker B. Elliott A. G. Lash L. H. Huang J. X. (2016). Activity and predicted nephrotoxicity of synthetic antibiotics based on polymyxin B. J. Med. Chem. 59, 1068–1077. 10.1021/acs.jmedchem.5b01593 26734854 PMC4774972

[B19] Hasan B. Perveen K. Olsen B. Zahra R. (2014). Emergence of carbapenem-resistant Acinetobacter baumannii in hospitals in Pakistan. J. Med. Microbiol. 63, 50–55. 10.1099/jmm.0.063925-0 24085817

[B20] Hasson S. S. Al-Balushi M. S. Alharthy K. Al-Busaidi J. Z. Aldaihani M. S. Othman M. S. (2013). Evaluation of anti-resistant activity of Auklandia (Saussurea lappa) root against some human pathogens. Asian pac. J. Trop. Biomed. 3, 557–562. 10.1016/S2221-1691(13)60113-6 23836413 PMC3695582

[B21] Hood M. I. Becker K. W. Roux C. M. Dunman P. M. Skaar E. P. (2013). genetic determinants of intrinsic colistin tolerance in Acinetobacter baumannii. Infect. Immun. 81, 542–551. 10.1128/IAI.00704-12 23230287 PMC3553813

[B22] Hsu L. Y. Apisarnthanarak A. Khan E. Suwantarat N. Ghafur A. Tambyah P. A. (2017). Carbapenem-resistant acinetobacter baumannii and enterobacteriaceae in South and southeast asia. Clin. Microbiol. Rev. 30, 1–22. 10.1128/CMR.00042-16 27795305 PMC5217790

[B23] Ibrahim S. Al-Saryi N. Al-Kadmy I. M. S. Aziz S. N. (2021). Multidrug-resistant Acinetobacter baumannii as an emerging concern in hospitals. Mol. Biol. Rep. 48, 6987–6998. 10.1007/s11033-021-06690-6 34460060 PMC8403534

[B45] Isler B. Doi Y. Bonomo R. A. Paterson D. L. (2019). New treatment options against Carbapenem-resistant Acinetobacter baumannii infections. Antimicrob. Agents Chemother. 63 (1). 10.1128/aac.01110-18 PMC6325237 30323035

[B25] Jacobo-Salcedo Mdel R. Gonzalez-Espindola L. A. Alonso-Castro A. J. Gonzalez-Martinez Mdel R. Domínguez F. Garcia-Carranca A. (2011). Antimicrobial activity and cytotoxic effects of Magnolia dealbata and its active compounds. Nat. Prod. Commun. 6, 1934578X1100600–4. 10.1177/1934578x1100600818 21922914

[B26] Karah N. Khalid F. Wai S. N. Uhlin B. E. Ahmad I. (2020). Molecular epidemiology and antimicrobial resistance features of Acinetobacter baumannii clinical isolates from Pakistan. Ann. Clin. Microbiol. Antimicrob. 19, 2. 10.1186/s12941-019-0344-7 31941492 PMC6964048

[B27] Kempf M. Rolain J.-M. (2012). Emergence of resistance to carbapenems in acinetobacter baumannii in europe: clinical impact and therapeutic options. Int. J. Antimicrob. Agents 39, 105–114. 10.1016/j.ijantimicag.2011.10.004 22113193

[B28] Khalid F. Saleem S. Ahmad I. (2020). High prevalence of carbapenem-resistant Acinetobacter baumannii associated respiratory tract infections in Pakistani hospitals. J. Pak. Med. Assoc. 70, 1630–1632. 10.5455/JPMA.35384 33040124

[B29] Koch E. Klaas C. A. Rüngeler P. Castro V. Mora G. Vichnewski W. (2001). Inhibition of inflammatory cytokine production and lymphocyte proliferation by structurally different sesquiterpene lactones correlates with their effect on activation of NF-kappaB. Biochem. Pharmacol. 62, 795–801. 10.1016/s0006-2952(01)00714-6 11551526

[B30] Larsen M. V. Cosentino S. Rasmussen S. Friis C. Hasman H. Marvig R. L. (2012). Multilocus sequence typing of total-genome-sequenced bacteria. J. Clin. Microbiol. 50, 1355–1361. 10.1128/JCM.06094-11 22238442 PMC3318499

[B31] Lewis K. Ausubel F. M. (2006). Prospects for plant-derived antibacterials. Nat. Biotechnol. 24, 1504–1507. 10.1038/nbt1206-1504 17160050

[B32] Mcconnell M. J. Actis L. Pachón J. (2013). Acinetobacter baumannii: human infections, factors contributing to pathogenesis and animal models. FEMS Microbiol. Rev. 37, 130–155. 10.1111/j.1574-6976.2012.00344.x 22568581

[B33] Moffatt J. H. Harper M. Harrison P. Hale J. D. Vinogradov E. Seemann T. (2010a). Colistin resistance in Acinetobacter baumannii is mediated by complete loss of lipopolysaccharide production. Antimicrob. Agents Chemother. 54, 4971–4977. 10.1128/AAC.00834-10 20855724 PMC2981238

[B34] Mu X. Wang N. Li X. Shi K. Zhou Z. Yu Y. (2016). The effect of colistin resistance-associated mutations on the fitness of acinetobacter baumannii. Front. Microbiol. 7, 1715. 10.3389/fmicb.2016.01715 27847502 PMC5088200

[B35] Paterson D. L. (2006). Resistance in gram-negative bacteria: Enterobacteriaceae. Am. J. Infect. Control 34, S20–S28. 10.1016/j.ajic.2006.05.238 16813978

[B36] Poirel L. Jayol A. Nordmann P. (2017). Polymyxins: antibacterial activity, susceptibility testing, and resistance mechanisms encoded by plasmids or chromosomes. Clin. Microbiol. Rev. 30, 557–596. 10.1128/CMR.00064-16 28275006 PMC5355641

[B37] Pournaras S. Gogou V. Giannouli M. Dimitroulia E. Dafopoulou K. Tsakris A. (2014). Single-locus-sequence-based typing of blaOXA-51-like genes for rapid assignment of Acinetobacter baumannii clinical isolates to international clonal lineages. J. Clin. Microbiol. 52, 1653–1657. 10.1128/JCM.03565-13 24622099 PMC3993655

[B38] Pyun H. Kang U. Seo E. K. Lee K. (2018). Dehydrocostus lactone, a sesquiterpene from Saussurea lappa Clarke, suppresses allergic airway inflammation by binding to dimerized translationally controlled tumor protein. Phytomedicine 43, 46–54. 10.1016/j.phymed.2018.03.045 29747753

[B39] Rao Vadaparthi P. R. Kumar K. Sarma V. U. Hussain Q. A. Babu K. S. (2015). Estimation of costunolide and dehydrocostus lactone in Saussurea lappa and its polyherbal formulations followed by their stability studies using HPLC-DAD. Pharmacogn. Mag. 11, 180–190. 10.4103/0973-1296.149736 25709231 PMC4329622

[B40] Rice L. B. (2008). Federal funding for the study of antimicrobial resistance in nosocomial pathogens: no ESKAPE. J. Infect. Dis. 197, 1079. 10.1086/533452 18419525

[B41] Thadtapong N. Chaturongakul S. Soodvilai S. Dubbs P. (2021)., Colistin and carbapenem-resistant acinetobacter baumannii Aci46 in Thailand: Genome analysis and antibiotic resistance profiling Antibiotics 10 (9). 1054. 10.3390/antibiotics10091054 34572636 PMC8468411

[B42] Tipton K. A. Dimitrova D. Rather P. N. (2015). Phase-variable control of multiple phenotypes in acinetobacter baumannii strain AB5075. J. Bacteriol. 197, 2593–2599. 10.1128/JB.00188-15 26013481 PMC4518826

[B43] Zhang Z. Schwartz S. Wagner L. Miller W. (2000). A greedy algorithm for aligning DNA sequences. J. Comput. Biol. 7, 203–214. 10.1089/10665270050081478 10890397

