# Supplementary material for: Emergence of high colistin resistance in carbapenem resistant Acinetobacter baumannii in Pakistan and its potential management through immunomodulatory effect of an extract from Saussurea lappa
Source: Front Pharmacol. 2022 Sep 16;13:986802. doi: 10.3389/fphar.2022.986802 (PMC9523213; doi:10.3389/fphar.2022.986802)
Supplement: Supplementary file 1 [file Table1.DOCX]

**Table S1: Primers used in the study**

| **Primers** | **Sequence 5'-3'** | **Product size** | **Reference** |
| --- | --- | --- | --- |
| oxa-51-like-F1 | TCCATACGGCAATTTCAACA | 1408 bp (IS*Aba1* not inserted) or 2597 bp (IS*Aba1* inserted) | (Karah et al., 2020) |
| oxa-51-like-R | GGCTTGACGCTGCTTTTTAC |  |  |

**Reference:**

KARAH, N., KHALID, F., WAI, S. N., UHLIN, B. E. & AHMAD, I. 2020. Molecular epidemiology and antimicrobial resistance features of Acinetobacter baumannii clinical isolates from Pakistan. *Ann Clin Microbiol Antimicrob,* 19**,** 2.
