# Supplementary figures and images for: Emergence of high colistin resistance in carbapenem resistant Acinetobacter baumannii in Pakistan and its potential management through immunomodulatory effect of an extract from Saussurea lappa
Source: Front Pharmacol. 2022 Sep 16;13:986802. doi: 10.3389/fphar.2022.986802 (PMC9523213; doi:10.3389/fphar.2022.986802)

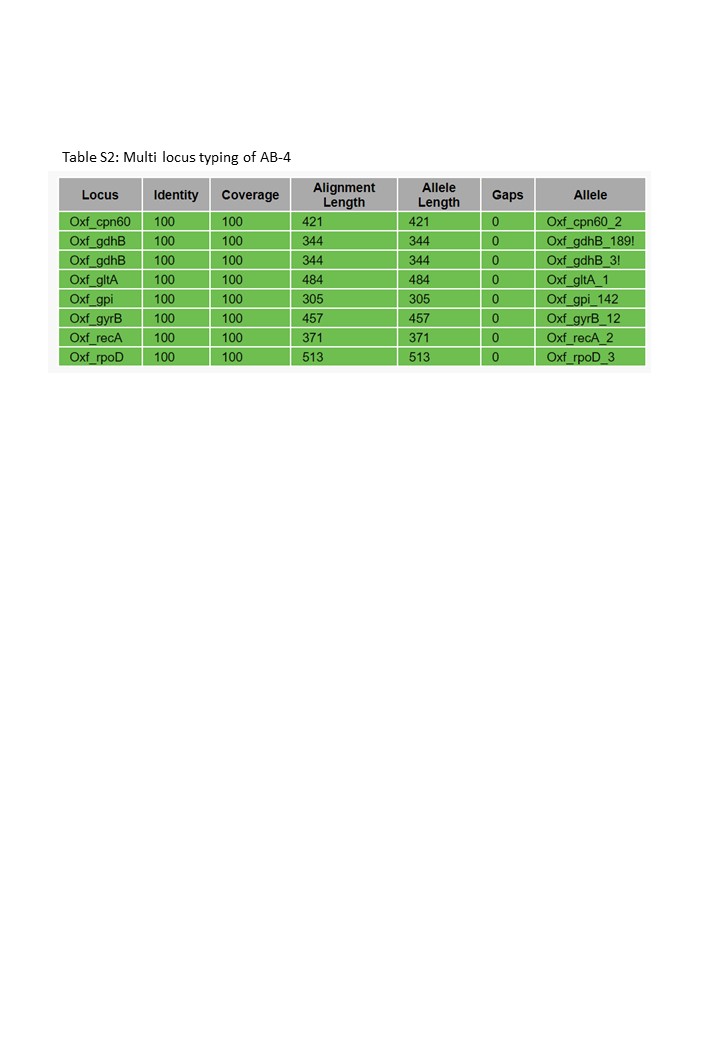

Supplement: Supplementary file 2 [file Image1.JPEG]

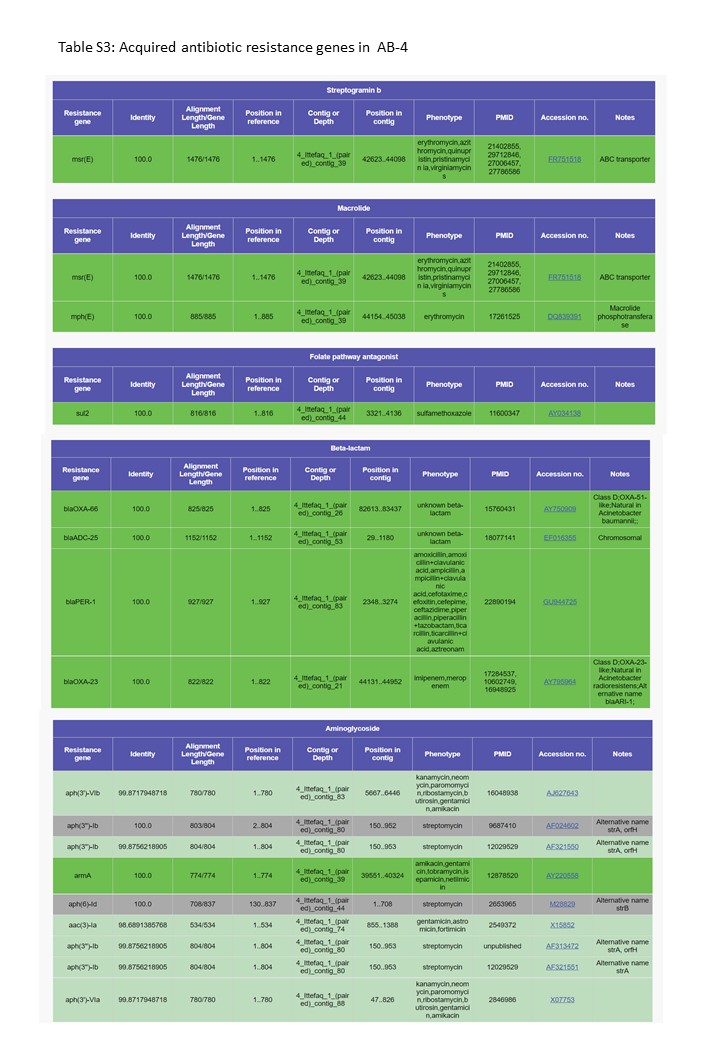

Supplement: Supplementary file 3 [file Image2.JPEG]
